# Beta-amyloid increases the expression level of ATBF1 responsible for death in cultured cortical neurons

**DOI:** 10.1186/1750-1326-6-47

**Published:** 2011-07-05

**Authors:** Cha-Gyun Jung, Kyung-Ok Uhm, Yutaka Miura, Takashi Hosono, Hirofumi Horike, Kum Kum Khanna, Mi-Jeong Kim, Makoto Michikawa

**Affiliations:** 1 Department of Alzheimer's Disease Research, Research Institute, National Center for Geriatrics and Gerontology (NCGG), 35, Morioka, Obu, Aichi 474-8511, Japan; 2 Department of Molecular Neurobiology, Graduate School of Medical Sciences, Nagoya City University, Nagoya, 467-8601, Japan; 3 Queensland Institute of Medical Research and University of Queensland Centre, for Clinical Research, Brisbane 4029, Queensland, Australia

## Abstract

**Background:**

Recently, several lines of evidence have shown the aberrant expression of cell-cycle-related proteins and tumor suppressor proteins in vulnerable neurons of the Alzheimer's disease (AD) brain and transgenic mouse models of AD; these proteins are associated with various paradigms of neuronal death. It has been reported that ATBF1 induces cell cycle arrest associated with neuronal differentiation in the developing rat brain, and that gene is one of the candidate tumor suppressor genes for prostate and breast cancers in whose cells overexpressed ATBF1 induces cell cycle arrest. However, the involvement of ATBF1 in AD pathogenesis is as yet unknown.

**Results:**

We found that ATBF1 was up-regulated in the brains of 17-month-old Tg2576 mice compared with those of age-matched wild-type mice. Moreover, our *in vitro *studies showed that Aβ1-42 and DNA-damaging drugs, namely, etoposide and homocysteine, increased the expression ATBF1 level in primary rat cortical neurons, whereas the knockdown of ATBF1 in these neurons protected against neuronal death induced by Aβ1-42, etoposide, and homocysteine, indicating that ATBF1 mediates neuronal death in response to these substances. In addition, we found that ATBF1-mediated neuronal death is dependent on ataxia-telangiectasia mutated (ATM) because the blockage of ATM activity by treatment with ATM inhibitors, caffeine and KU55933, abolished ATBF1 function in neuronal death. Furthermore, Aβ1-42 phosphorylates ATM, and ATBF1 interacts with phosphorylated ATM.

**Conclusions:**

To the best of our knowledge, this is the first report that Aβ1-42 and DNA-damaging drugs increased the ATBF1 expression level in primary rat cortical neurons; this increase, in turn, may activate ATM signaling responsible for neuronal death through the binding of ATBF1 to phosphorylated ATM. ATBF1 may therefore be a suitable target for therapeutic intervention of AD.

## Background

Alzheimer's disease (AD), a progressive neurodegenerative disorder of the elderly, is associated with a chronic loss of synapses and neuronal death, and is characterized by the presence of parenchymal deposits of amyloid-β peptides (Aβ), the major protein component of senile plaques [1,2]. Accumulation of Aβ in the brain is associated with disease-causing inherited variants of the amyloid precursor protein (APP) [3], presenilin 1 (PS1) [4], presenilin 2 (PS2) [5], and apoplipoprotein E (APOE) [6] genes, and an increased extracellular Aβ level is a major cause of neuronal death in AD. In addition to genetic evidence that Aβ promotes neuronal degeneration and death *in vivo *[7,8], *in vitro *studies show that Aβ aggregates rapidly induce neuronal death by necrosis or apoptosis [9,10], and Aβ-induced neurotoxicity involves oxidative stress, inflammation, and perturbation of calcium homeostasis [1]. However, the mechanisms by which neuronal degeneration and death occur in AD and whether they are induced by Aβ are not completely understood.

One focus in the mechanism of neuronal death in AD is the aberrant expression of cell-cycle-related proteins, such as cdc2, cdk4, cyclin B1, and cyclin D, which mediate cell cycle progression, in vulnerable neurons of the AD brain [11-14]; these molecules play essential roles in neuronal death associated with various paradigms of neuronal death [15]. In addition to cell cycle progression molecules, a number of cell cycle inhibitors, such as p16 and p27 [13,16], and tumor suppressor proteins such as p53 and BRCA1 [17,18] are also increased in levels in the AD brain. In addition to the human AD brain, the increased expression levels of cell-cycle-related proteins were also found in transgenic mouse models of AD [19,20]. Although it is unclear why cell-cycle-related proteins show increased in levels in the AD brain and AD mouse models, one possibility is that DNA damage induced by Aβ may increase the levels of or activate these molecules. Indeed, DNA damage was found in the AD brain, and Aβ increases Cdc25A [21], Cdk4, and p53 [22] levels in primary rat neurons resulting in neuronal death. Recently, Kruman et al. have reported that cultured postmitotic cortical neurons exposed to Aβ undergo apoptosis that is dependent on tumor suppressor factor ataxia-telangiectasia mutated (ATM) activity, whereas treatment with caffeine, which is an ATM inhibitor, can exert a neuroprotective effect on cultured neurons exposed to Aβ [22]. In this context, ATM appears to potentiate neuronal apoptosis.

AT-motif binding factor 1 (ATBF1) is a 404 kDa transcription factor that contains 4 homeodomains and 23 zinc-finger motifs [23] involved in transcription regulations and protein-protein interactions [24]. We previously reported that ATBF1 is highly expressed in postmitotic neurons but not in neural progenitor cells, and it induces cell cycle arrest associated with neuronal differentiation in the developing rat brain [25]. We also found that sublocalization of ATBF1 is regulated by phospatidylinositol-3 (PI3) kinase including ATM [25], indicating that ATBF1 is one of the targets of ATM. Indeed, ATM phosphorylates ATBF1 at Ser1180 in HEK293T cells exposed to 10-Gy radiation [26]. ATBF1 also interacts with p53 to activate the p21^Waf1/Cip1 ^promoter to trigger cell cycle arrest [27]. It has also been reported that the ATBF1 gene is one of the candidate tumor suppressor genes for prostate and breast cancers in whose cells overexpressed ATBF1 induces cell cycle arrest [28,29]. However, the involvement of ATBF1 in AD pathogenesis is as yet unknown.

In this study, we investigated whether ATBF1 expression is altered in the brains of Tg2576 mice similarly to other cell-cycle-related molecules, and we found an up-regulated ATBF1 expression in the brains of Tg2576 mice compared with those of age-matched wild-type mice. Moreover, our *in vitro *studies showed that Aβ and DNA-damaging drugs, namely, etoposide and homocysteine, increased the ATBF1 expression level in primary rat cortical neurons; this increase, in turn, may activate ATM signaling responsible for neuronal death through the binding of ATBF1 to phosphorylated ATM.

## Results

### ATBF1 was up-regulated in the brains of 17-month-old Tg2576 mice compared with those of age-matched wild-type mice

**Figure 1:**
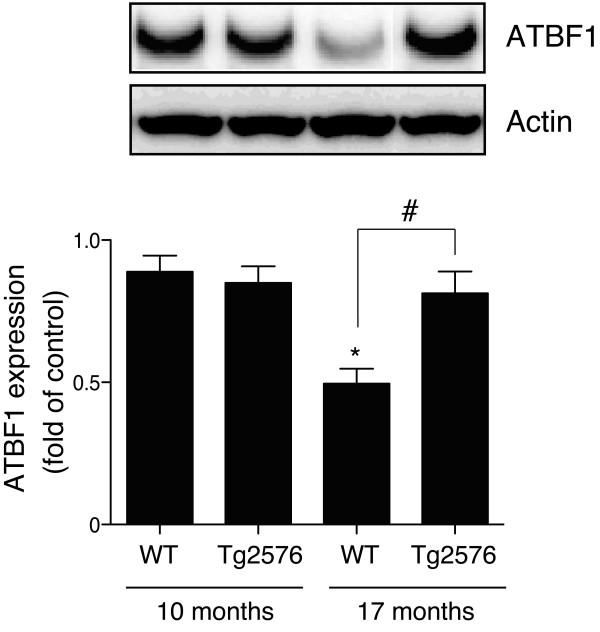
**ATBF1 protein expression level appears to decrease in age-dependent manner in wild-type mice**. It increased in the brains of 17-month-old Tg2576 mice compared with age-matched wild-type mice. Lysates were prepared from whole brains of 10- and 17-month-old Tg2576 (*n *= 6) and age-matched wild-type (WT) mice (*n *= 6). The expression level of ATBF1 was determined by Western blot analysis using the anti-ATBF1 antibody. A, representative immunoblot is shown (upper panel), and bands were quantified by densitometry, normalized to actin, and expressed as a value relative to that in the 10-month-old WT mice as a control (lower graph). All the values are presented as mean ± SEM. *p < 0.05 *vs *10-month-old WT mice (n = 6) and #*p *< 0.05 compared with those of 17-month-old WT mice, as determined by one-way ANOVA followed by Duncan's test.

We first investigated whether ATBF1 expression is altered in the brains of Tg2576 mice overexpressing human APP with the Swedish mutation. Total proteins were extracted from whole brains of 10- and 17-month-old Tg2576 and age-matched wild-type mice, and subjected to Western blot analysis. We found that the ATBF1 expression level in the brains of 17-month-old wild-type mice was lower than that in the brains of 10-month-old wild-type mice. However, ATBF1 expression was significantly up-regulated in 17-month-old Tg2576 mice compared with age-matched wild-type mice, whereas there was no significant difference between Tg2576 and wild-type mice at the age of 10 months (Figure 1).

### Aβ1-42 and DNA-damaging drugs, etoposide and homocysteine, increased ATBF1 expression level in cultured rat cortical neurons

**Figure 2:**
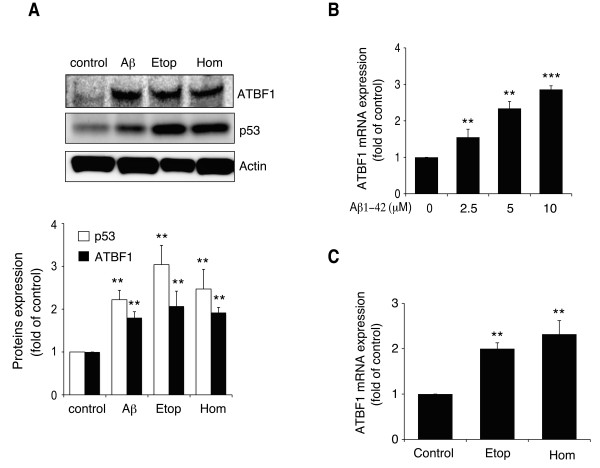
**ATBF1 protein and mRNA expression levels increased in primary cortical neurons after treatment with Aβ1-42 and DNA-damaging drugs, namely, etoposide (Etop) and homocysteine (Hom)**. A, primary cortical neurons were seeded at a density of 1 × 10^6 ^cells/ml in poly-d-lysine-coated 60 mm culture dishes. Three days after plating, the cells were treated for 16 h with 10 μM Aβ1-42, 1 μM etoposide, or 250 μM homocysteine, and the cells were then harvested. The expression levels of ATBF1 and p53 were determined by Western blot analysis using the anti-ATBF1 and anti-p53 antibodies. A, a representative immunoblot is shown (upper panel), and bands were quantified by densitometry, normalized to actin, and expressed as a value relative to that of the control (lower panel). B and C, primary cortical neurons were treated for 16 h with 0, 2.5, 5, or 10 μM Aβ1-42 (B), or 250 μM homocysteine or 1 μM etoposide (C). ATBF mRNA expression level was determined by real-time PCR analysis. The expression level of ATBF1 mRNA was normalized to the corresponding amount of actin mRNA and expressed as a value relative to that of the control. All the values are presented as the mean ± SEM of three independent experiments. *p < 0.05, **p < 0.01, ***p < 0.001 *vs *control, as determined by Student's *t*-test.

In Tg2576 brains, the accumulation of Aβ occurs from 15 to 23 months but is not observed in appreciable amounts until 12 months [30]. Therefore, we hypothesized that an increase in ATBF1 expression level in the brains of 17-month-old Tg2576 mice is due to an increase in Aβ level. To test this hypothesis, we determined by Western blot analysis the protein expression levels of ATBF1 and p53, which play a key role in the regulation of cell viability in response to DNA-damaging drugs in many cell types including neurons, in cultured rat cortical neurons treated with 10 μM Aβ1-42 for 16 h. The Aβ1-42 peptide used in our experiments was largely monomer (see "Additional file 1"). We observed that Aβ1-42 significantly increased ATBF1 and p53 protein expression levels in these cells (Figure 2A). A previous study showed that the expression level of ATBF1 is increased in gastric cancer cells exposed to mitomycin-C, which can induce DNA damage in many cell types [31]. This finding suggests that DNA damage might increase ATBF1 expression level because Aβ can also induce neuronal apoptosis through oxidative DNA damage. Therefore, we treated cultured cortical neurons with two different DNA-damaging drugs, etoposide and homocysteine, which are used commonly as DNA-damaging drugs for many cells types including neurons, and we found that these two drugs significantly increased ATBF1 and p53 protein expression levels (Figure 2A). Next, we measured the expression levels of ATBF1 mRNA in cultured cortical neurons treated with Aβ1-42 at an indicated dose by semiquantitative real-time PCR analysis. As shown in Figure 2B, treatment with Aβ1-42 significantly increased ATBF1 mRNA expression level in a dose-dependent manner compared with the control, and etoposide and homocysteine also increased ATBF1 mRNA expression level (Figure 2C). These findings indicate that an increase in ATBF1 protein expression level induced by Aβ1-42, etoposide, and homocysteine is caused by an increase in ATBF1 gene expression level.

### Knockdown of ATBF1 in cultured cortical neurons protected against Aβ1-42-, etoposide-, and homocysteine-induced neurotoxicity

**Figure 3:**
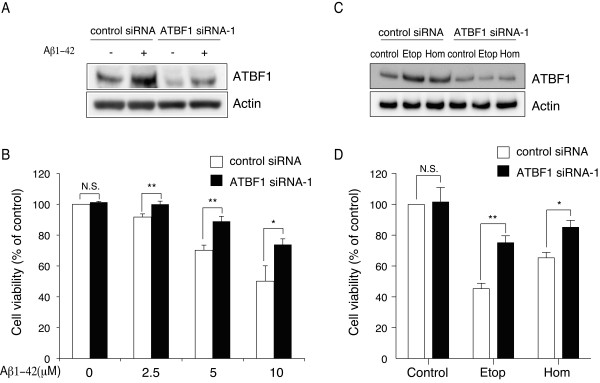
**ATBF1 knockdown decreases the viability of primary cortical neurons upon treatment with Aβ1-42, etoposide (Etop), or homocysteine (Hom)**. A and C, knockdown of ATBF1 in primary cortical neurons. Primary cortical neurons were transfected with ATBF1 siRNA-1 or control siRNA for 48 h. After transfection, the cells were then incubated in the presence or absence of 10 μM Aβ1-42 (A), 1 μM etoposide (C), or 250 μM homocysteine (C) for 16 h. The expression levels of ATBF1 and actin were determined by Western blot analysis using the anti-ATBF1 and anti-actin antibodies. B and D, primary cortical neurons were seeded at a density of 1 × 10^6 ^cells/ml in poly-d-lysine-coated 96-well plates. Three days after plating, cells were transfected with ATBF1 siRNA-1 or control siRNA for 48 h, and the cells were then treated with 0, 2.5, 5, or 10 μM Aβ1-42 (B), 1 μM etoposide (D), or 250 μM homocysteine (D) for 16 h. Cell viability was determined using a CellTiter-Glo luminescent cell viability assay kit and is shown as a percentage of surviving cells. All the values are presented as the mean ± SEM of three independent experiments. *p < 0.05, **p < 0.01 *vs *control siRNA treatment. N.S., not significant, as determined by Student's *t*-test.

Aβ1-42, etoposide, and homocysteine induce death of cultured cortical neurons *in vitro *[22]. Next, we examined whether ATBF1 mediates neuronal death after treatment with Aβ1-42, etoposide, and homocysteine. For this purpose, we first decreased the ATBF1 expression level in primary cortical neurons by ATBF1 siRNA transfection. The cells were transfected with ATBF1 siRNA-1 or control siRNA, as described in "Experimental Procedures". Forty-eight hours after transfection, the ATBF1 protein expression level was determined by Western blot analysis using an anti-ATBF1 antibody. As shown in Figures 3A and 3C, the transfection of ATBF1 siRNA-1 decreased the ATBF1 protein level by about 75% in cultured cortical neurons compared with control siRNA transfection. This finding indicates that endogenous ATBF1 can be efficiently knocked down in these cells by transfection of ATBF1 siRNA-1. Next, we determined the effects of ATBF1 knockdown on neuronal survival against Aβ1-42-, etoposide-, and homocysteine-induced neurotoxicity. Cultured cortical neurons transfected with ATBF1 siRNA-1 or control siRNA were treated with Aβ1-42 at an indicated dose, 1 μM etoposide, or 250 μM homocysteine for 16 h. Cell viability was then assessed using a CellTitle-Glo luminescent cell viability assay kit. We were able to detect differences in cell viability only by ATBF1 siRNA-1 transfection compared with control siRNA transfection. The percentage of surviving neurons decreased in control-siRNA-transfected cells after the treatment with Aβ1-42, etoposide, or homocysteine. However, the percentage of surviving neurons increased in ATBF1-siRNA-1-transfected cells compared with control-siRNA-transfected cells after the treatment with Aβ1-42 (Figure 3B), etoposide, or homocysteine (Figure 3D). These findings indicate that ATBF1 could mediate neuronal death in response to the treatment with Aβ1-42, etoposide, or homocysteine. We also determined the effects of another ATBF1 siRNA (ATBF1-siRNA-2) on neuronal survival against Aβ1-42-induced neurotoxicity, and obtained similar result (Additional file 2). Therefore, we used ATBF1 siRNA-1 to ATBF1 knockdown for the following experiments.

### ATBF1 mediated apoptotic function in cultured cortical neurons against Aβ1-42-induced neurotoxicity

**Figure 4:**
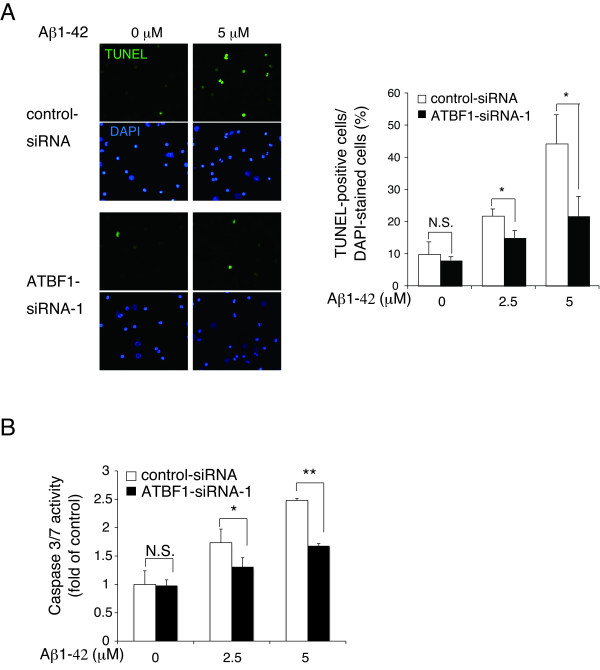
**ATBF1 regulates apoptosis in primary cortical neurons in the presence of Aβ1-42-induced neurotoxicity**. A, primary cortical neurons were seeded at a density of 1 × 10^6 ^cells/ml in poly-d-lysine-coated 24-well glass plates. Three days after plating, the cells were transfected with ATBF1 siRNA-1 or control siRNA for 48 h. After transfection, the cells were then incubated with 0, 2.5, or 10 μM Aβ1-42 for 16 h, and apoptosis was analyzed by TUNEL assay. Cell nuclei were also stained with DAPI. Left panel: Representative images showing DAPI-stained and TUNEL-positive cells. Right panel: quantification of TUNEL. TUNEL-positive neurons were counted from at least 400 neurons from five randomly selected fields in three independent experiments. B, primary cortical neurons were seeded at a density of 1 × 10^6 ^cells/ml in poly-d-lysine-coated 96-well plates. Three days after plating, cells were transfected with ATBF1 siRNA-1 or control siRNA for 48 h, and the cells were then treated with 0, 2.5, or 5 μM Aβ1-42 for 16 h. Caspase-3/7 activity was determined using a Caspase-Glo™ 3/7 assay kit. All the values are presented as the mean ± SEM of three independent experiments. *p < 0.05, **p < 0.01 *vs *control siRNA. N.S., not significant, as determined by one-way ANOVA followed by Duncan's test.

To determine whether apoptosis is responsible for the survival of cultured cortical neurons with decreased ATBF1 expression levels, we analyzed DNA breaks by terminal deoxynucleotidyl transferase-mediated dUTP nick-end labeling (TUNEL) assay of ATBF1-siRNA- and control-siRNA-transfected cells after Aβ1-42 treatment. Figure 4A shows representative images of TUNEL-positive cells and total nuclei. The treatment of control siRNA-transfected cells with Aβ1-42 resulted in a significant increase in the number of TUNEL-positive cells compared with nontreatment (Figure 4A). However, the percentage of TUNEL-positive cells among ATBF1-siRNA-transfected cells treated with Aβ1-42 was lower than that among control-siRNA-transfected cells (Figure 4A), indicating that the knockdown of ATBF1 significantly reduced the extent of Aβ1-42-induced apoptosis. The knockdown of ATBF1 alone showed no significant increase in the percentage of TUNEL-positive cells (Figure 4A). To confirm these findings, we performed a similar experiment, and caspase-3/7 activity was determined using a Caspase-Glo 3/7 assay kit. It has been reported that Aβ may lead to the induction of caspase-3-mediated pathways that are involved in oxidative stress [32]. The treatment of control siRNA-transfected cells with Aβ1-42 increased the activity of caspase-3/7 compared with nontreatment (Figure 4B). However, a decreased activity of caspase-3/7 was detected in ATBF1-siRNA-transfected cells treated with Aβ1-42, indicating that ATBF1 is at least one vital component for the activation of caspase-3/7 in cultured cortical neurons after Aβ1-42 treatment.

### Overexpression of ATBF1 itself in primary cortical neurons did not induce apoptosis

**Figure 5:**
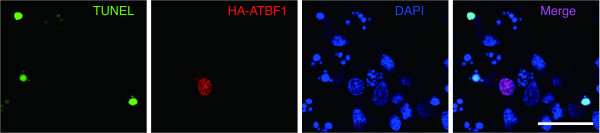
**Overexpression of HA-tagged ATBF1 itself in primary cortical neurons did not induce apoptosis**. TUNEL of primary cortical neurons transfected with HA-tagged ATBF1. The cells were transiently transfected with HA-tagged ATBF1. Twenty-four hours after transfection, TUNEL was performed, and then the cells were stained with the anti-HA antibody to detect transfected HA-ATBF1. Scale bars: 25 μm.

Next, we examined whether overexpression of ATBF1 itself induces apoptosis in cultured cortical neurons. The cells were transfected with HA-tagged full-length human ATBF1 cDNA. Twenty-four hours after transfection, we performed TUNEL assay, and then counted TUNEL-positive cells among HA-ATBF1-transfected cells. We found that cells transfected with HA-ATBF1 were largely TUNEL-negative (95.2 ± 1.2%) (Figure 5). This finding is consistent with our previous finding that overexpression of ATBF1 in Neuro 2A cells (mouse neuroblastoma cell line) by transfection of the HA-ATBF1 expression vector did not induce apoptosis [25].

### ATBF1-mediated neuronal death after Aβ1-42 treatment depended on ATM

**Figure 6:**
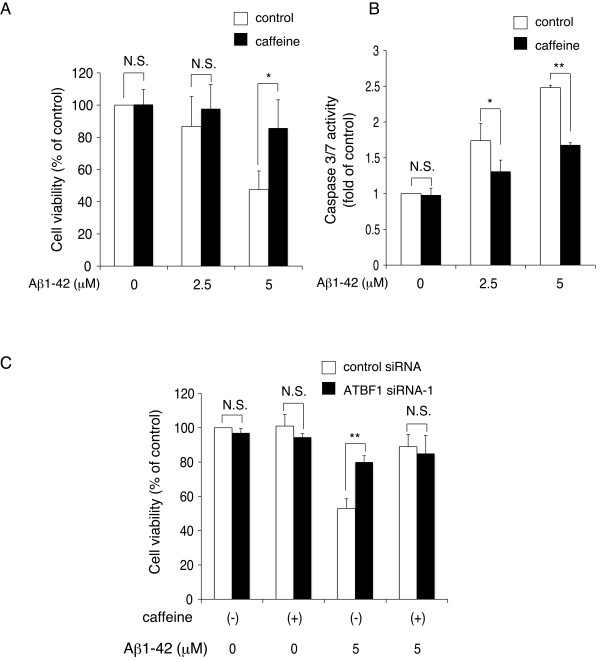
**A and B, caffeine, an inhibitor of ATM, shows a neuroprotective effect against Aβ-induced neurotoxicity**. A and B, primary cortical neurons were seeded at a density of 1 × 10^6 ^cells/ml in poly-d-lysine-coated 96-well plates. Three days after plating, cells were pretreated with 10 μM caffeine for 1 h, and subsequently treated for 16 h with 0, 2.5, or 5 μM Aβ1-42. Cell viability was determined using a CellTiter-Glo luminescent cell viability assay kit (A), and caspase-3/7 activity was determined using a Caspase-Glo™ 3/7 assay kit (B). C, after transfection as described in Fig 3B, cells were pretreated for 1 h with or without 10 μM caffeine, and then cells were further incubated with or without 5 μM Aβ1-42 for 16 h. Cell viability was determined using a CellTiter-Glo luminescent cell viability assay kit. All the values are presented as the mean ± SEM of three independent experiments. *p < 0.05, **p < 0.01 *vs *control siRNA. N.S., not significant, as determined by one-way ANOVA followed by Duncan's test.

Recent findings have shown that the ATM signaling pathway is essential for Aβ-induced neuronal death *in vitro *and *in vivo*, and treatment with caffeine, an inhibitor of ATM, protects cultured cortical neurons against apoptosis induced by Aβ1-42 [22]. Our previous data have shown that the nuclear localization of ATBF1 is suppressed by treatment with caffeine, indicating that ATBF1 function could be regulated by ATM [25]. Moreover, it has also been reported that the ATBF1 gene is one of the target genes of ATM, which phosphorylates ATBF1 at Ser1180 [26]. Therefore, we examined whether ATBF1-mediated neuronal death after Aβ1-42 treatment is dependent on ATM. To determine whether caffeine can protect against neuronal death induced by Aβ1-42, we analyzed the effects of caffeine on cell viability (Figure 6A) and caspase-3/7 activity (Figure 6B). Cultured cortical neurons were pretreated with 10 μM caffeine for 1 h and subsequently treated with 2.5 μM and 5 μM Aβ1-42 for 16 h. The cells were then assessed for cell viability and caspase-3/7 activity using CellTitle-Glo luminescent cell viability assay and Caspase-Glo™ 3/7 assay kits, respectively. As shown in Figures 6A and 6B, treatment with caffeine decreased the number of dead cells treated with Aβ1-42 (Figure 6A) and decreased caspase-3/7 activity (Figure 6B) compared with the nontreatment control. We also tested the effect of KU55933, a specific inhibitor of ATM [33], on cell viability. As shown in Additional file 3A, treatment with KU55933 decreased the number of dead cells treated with Aβ1-42, etoposide, or homocysteine at concentration as low as 1 μM. These findings indicated that treatment with ATM inhibitors protect against Aβ1-42-, etoposide-, or homocysteine-induced neuronal death. Next, we assessed the effect of siRNA-mediated ATBF1 knockdown on Aβ1-42-induced neuronal death after treatment with caffeine or KU55933. As shown in Figure 6C and Additional file 3, there are no significant differences in the percentage of survival between ATBF1 siRNA-transfected neurons with treatment of caffeine or KU55933 and those without treatment with caffeine or KU55933. These findings indicate that ATBF1 is required for neuronal death in response to Aβ1-42 treatment, which could be dependent on ATM signaling.

### ATBF1 interacted with phosphorylated ATM

**Figure 7:**
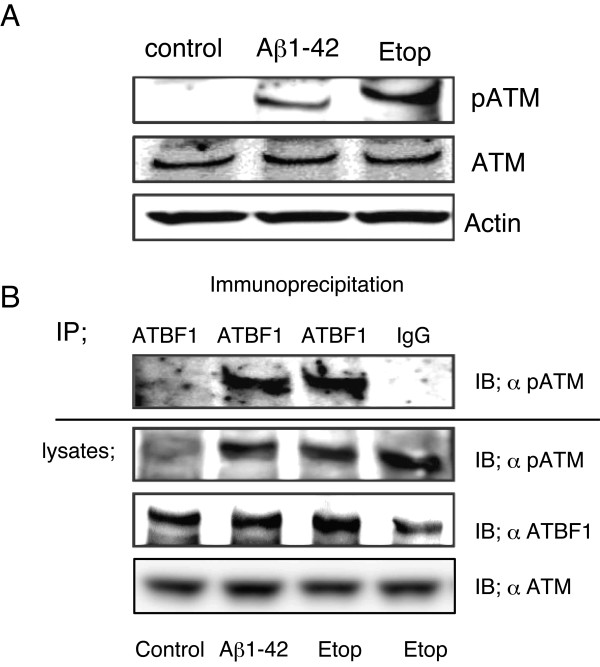
**A, phosphorylation of ATM after treatment of primary cortical neurons with Aβ1-42 or etoposide**. Primary cortical neurons were treated for 1 h with 10 μM Aβ1-42 or with 1 μM etoposide (Etop). The cells were lysed, and the protein expression levels of pATM, ATM, and actin were determined by Western blot analysis using the anti-pATM, anti-ATM, and anti-actin antibodies. B, ATBF1 interacts with pATM after treatment with Aβ1-42 or etoposide. Cultured cortical neurons were treated as described in A, cell lysates were immunoprecipitated with the anti-ATBF1 antibody. The immunoprecipitated complex was then subjected to immunoblotting with the anti-pATM at Ser1891 antibody. Typical bands representative of three independent experiments with similar results are shown.

It is not known whether Aβ1-42 can induce the phosphorylation of ATM in cultured cortical neurons. We therefore analyzed the effect of Aβ1-42 on the expression level of phosphorylated ATM (pATM) at Ser1981, as an indicator of ATM activation, in cultured cortical neurons. Cultured cortical neurons were treated with 10 μM Aβ1-42 for 3 h or with 1 μM etoposide for 1 h as the positive control, and pATM expression level was determined by Western blot analysis using a specific antibody to ATM at Ser1981. We found an increase in pATM levels after the treatments with Aβ1-42 and etoposide (Figure 7A). To determine whether ATBF1 interacts with pATM, coimmunoprecipitation analysis was performed. Cultured cortical neurons were treated with 10 μM Aβ1-42 for 3 h or 1 μM etoposide for 1 h, and then subjected to immunoprecipitation with anti-ATBF1 antibody-conjugated Protein G beads followed by immunoblotting with the anti-pATM antibody. As shown in Figure 7B, ATBF1 interacted with pATM after treatment with Aβ1-42 or etoposide. Our findings suggest that ATBF1 expression was enhanced by Aβ1-42 and DNA-damaging drugs (etoposide and homocysteine) and increased the expression level of ATBF1, which in turn activated ATM signaling responsible for neuronal death through the binding of ATBF1 to pATM.

### ATM was required for ATBF1 to activate the p21 promoter

**Figure 8:**
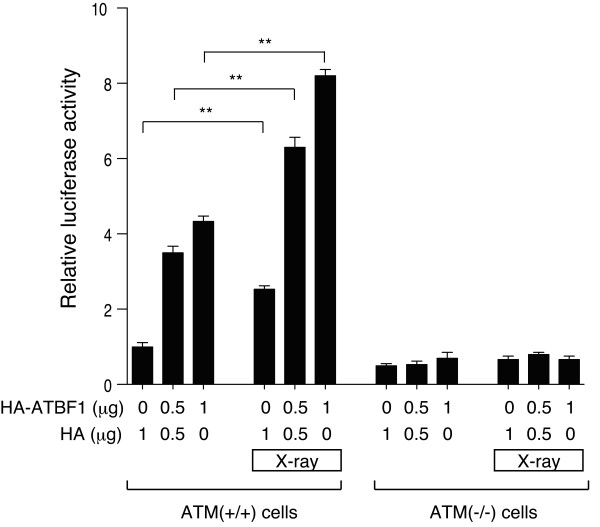
**Effect of ATBF1 on activation of p21 promoter in X-ray-irradiated ATM (+/+) and ATM (-/-) cells**. ATM (+/+) and ATM (-/-) cells were transfected with p21 promoter-luciferase, pRL-TK-luciferase (as an internal control), and an indicated dose of HA-ATBF1 vector or pCI-HA vector as a control. After 24 h, cells were irradiated with a 2.5 Gy X-ray, and then luciferase activity was measured after 12 h. All the values are presented as the mean ± SEM of three independent experiments. **p < 0.01 *vs *not irradiated. N.S., not significant, as determined by Mann-Whitney *U*-test with Bonferroni Correction.

To determine the functional relationship between ATBF1 and ATM, we carried out p21 (Waf1/Cip1) promoter assay using ATM (+/+) and ATM (-/-) human fibroblast cells. ATM has been shown to play a role in the induction of DNA double strand breaks to arrest the cell cycle via activation of p53, and ATBF1 activates the p21 promoter in collaboration with p53 [27]. As shown in Figure 8, irradiation with X-ray increased the p21 promoter activity in ATM (+/+) cells, but not in ATM (-/-) cells, which is consistent with a previous finding that p21 expression is not changed in ATM (-/-) cells treated with the DNA-damaging drug etoposide [34]. Overexpression of ATBF1 increased the p21 promoter activity in ATM (+/+) cells, but not in ATM (-/-) cells. The combination of X-ray irradiation and overexpression of ATBF1 in ATM (+/+) cells synergistically increased p21 promoter activity. Importantly, this effect of ATBF1 on p21 promoter activity was abolished in ATM (-/-) cells. This finding indicates that ATBF1 increases p21 promoter activity in an ATM-dependent manner.

## Discussion

Recently, cell-cycle-related molecules have been implicated as required components in the mechanisms underlying neuronal death in response to injury, stroke, and neurodegenerative diseases including AD [35-38] and transgenic mouse models of AD [19,20]. We have previously reported that ATBF1 is highly expressed in postmitotic neurons but not in neural progenitor cells in the developing rat brain, and that its mRNA expression level is highest in the embryonic day 12.5 (E12.5) brain [25]. Moreover, the overexpression of ATBF1 induces cell cycle arrest in mouse neuroblastoma, human prostate cancer, and human breast cancer cell lines [25,28,29]. These findings suggest that ATBF1 may play critical roles in cell cycle arrest and proliferation. In the present study, we found that the ATBF1 expression level in the brains of 17-month-old wild-type mice decreased compared with that in the brains of 10-month-old wild-type mice. This finding is consistent with our previous finding that ATBF1 mRNA expression level gradually decreases with increasing age in the rat brain [25]. However, ATBF1 expression was up-regulated in the brains of 17-month-old Tg2576 mice compared with that in the brains of age-matched wild-type mice. In Tg2576 mice, diffuse plaques appear after 12 months, and their amount gradually increases with age [30]. Therefore, we considered that the increase in ATBF1 expression level was due to Aβ, and we found that the treatment with Aβ1-42 significantly increased the expression levels of ATBF1 mRNA and protein in cultured rat cortical neurons. The increase in ATBF1 expression level in the brains of 17-month-old Tg2576 mice could be triggered by the accumulation of extracellular Aβ similar to the Aβ-mediated increase in ATBF1 expression level observed in cultured cortical neurons. In addition, the reason why ATBF1 remains increased in 17-month-old Tg2576 mice could be that Aβ induces neurons to re-enter the cell cycle and ATBF1 prevents this process from occurring.

Aβ induces oxidative DNA damage. A previous study showed that the expression level of ATBF1 is increased in gastric cancer cells treated with mitomycin-C, which can induce DNA damage in many cell types [31]. This suggests that DNA damage might increase ATBF1 expression level. We, therefore, also examined whether treatment with DNA-damaging drugs, namely, etoposide and homocysteine, affects ATBF1 expression. Here, we found that these DNA-damaging drugs significantly increased the expression levels of ATBF1 mRNA and protein in cultured rat cortical neurons. These findings suggest that the up-regulated ATBF1 expression observed in our *in vivo *and *in vitro *experiments could be due to DNA damage induced by Aβ.

It has been reported that the consequences of DNA damage are the expression of cell-cycle-related proteins [22,39,40] and activation of the family of phosphatidylinositol-3 (PI3)-kinases that include the ATM protein, which is involved in the regulation of cell cycle and apoptosis by the phosphorylation of many downstream substrates [41-43]. Therefore, one possibility is that ATM could constitute a common pathway activated in neuronal apoptosis after DNA damage. Recently, we have found that ATM induces ATBF1 expression during retinoicacid-induced neuronal differentiation of P19 cells by the activation and binding of CREB to a CRE consensus site located in the ATBF1 promoter (unpublished data). It has also been reported that the ATBF1 gene is one of the target genes of ATM that phosphorylates ATBF1 at Ser1180 [26]. These observations suggest that the activation of ATM highly correlates with the function and expression of ATBF1 as a gene regulatory factor. In this study, we observed that treatment with Aβ1-42 and etoposide rapidly posphorylates ATM at Ser 1981, and that ATBF1 interacts with pATM in cultured cortical neurons. Taken together, ATM activation induced by Aβ and DNA-damaging drugs may induce ATBF1 expression.

In this study, we also examined the effect of ATBF1 on neuronal death and apoptosis induced by Aβ1-42, etoposide, and homocysteine in cultured cortical neurons, and we found that the knockdown of ATBF1 by ATBF1 siRNA transfection significantly reduced the extent of cell death and apoptosis induced by Aβ1-42, etoposide, homocysteine. In addition, the knockdown of ATBF1 attenuated the activation of caspase-3/7. These findings suggest that the increased ATBF1 expression level may mediate apoptotic function in cultured cortical neurons against Aβ1-42-induced neurotoxicity. It has been reported that Aβ and DNA-damaging drugs induce the expression and activation of p53 which plays an important role in promoting apoptosis in cultured neurons [22,44]. Therefore, the increased ATBF1 expression level might simultaneously activate p53 to promote cell death, because ATBF1 interacts with p53 [27]. We also found in this study that ATBF1-mediated neuronal death is dependent on ATM signals because the blockage of ATM by treatment with ATM inhibitors, caffeine and KU55933, abolished ATBF1 functions in neuronal death. This finding is in agreement with our previous finding that caffeine treatment inhibits the translocalization of ATBF1 to the nucleus in P19 cells [25]. Further studies are necessary to characterize the role of ATBF1 in AD pathogenesis such as whether ATBF1 expression is altered in the AD brain.

## Conclusions

In conclusion, the increase in ATBF1 expression level observed in the brain of 17-month-old Tg2576 mice compared with age-matched wild-type mice could be caused by DNA damage induced by Aβ1-42, which in turn activates the ATM signaling responsible for neuronal death, indicating that ATBF1 plays an important role in neuronal death in response to Aβ1-42, etoposide, and homocystein, and it may be a useful target in the development of drugs to suppress the neuronal death induced by Aβ1-42.

## Methods

### Tg2576 mice

Female Tg2576 mice, an animal model of amyloid deposition, overexpressing human APP695 with the Swedish mutation K670N/M671, were obtained from Taconic (Germantown, NY). All the experiments were performed in accordance with the Guidelines for Animal Experiments of the Animal Experimentation Committee of the National Center for Geriatrics and Gerontology.

### Cell cultures

Cerebral cortical neurons were obtained from E17 Sprague-Dawley rats and cultured as described previously [45]. Briefly, embryonic brains were dissected, stripped of meninges, and minced with forceps. The minced tissue was incubated in 0.25% trypsin and 2 mg/ml DNase I in phosphate-buffered saline (PBS) at 37°C for 15 min. The fragments were then dissociated into single cells by pipetting. The dissociated cells were suspended in DMEM/F-12 medium (50:50%) containing N_2 _supplements and 7.5% bovine albumin fraction V, and plated onto poly-d-lysine-coated 60 mm dishes at a density of 1 × 10^6^/ml. These cells were used on day 4 of plating for further experiments. The immortalized fibroblast cell line AT22IJE-T was originally established from primary ataxia-telangiectasia (A-T) patient fibroblasts [46]. The cells were transfected with either the pEBS7 or pEGS7-YZ ATM vector to obtain AT22IJE-T/pEBS7 (ATM -/-) and AT22IJE-T/YZ5 (ATM +/+) cells, respectively [47]. Cells were maintained in DMEM containing 15% fetal bovine serum (FBS), 2 mM glutamine, 100 μg/ml hygromycin B, 100 U/ml penicillin, and 0.1 mg/ml streptomycin.

### RNA extraction and real-time PCR

Total RNA was isolated from primary cortical neurons using an RNeasy plus mini kit (Qiagen, Valencia, CA) following the manufacturer's instructions. Reverse transcription was performed using 1 μg of total RNA using a PrimeScript RT reagent kit (Takara, Tokyo, Japan). Real-time PCR was carried out using the SYBR Premix Ex Taq system and Thermal Cycler Dice Real-Time system (Takara). The expression of the ATBF1 gene was normalized with the corresponding amount of actin mRNA using the comparative threshold cycle method following the manufacturer's protocols. Amplification was performed using the following primers (sense and antisense): ATBF1 (5'-CAAAACTTCTGCTGCCCTTC-3' and 5'-GGCTTGTCTCAAGGTGC-TTC-3') and actin (5'-CATCCGTAAAGACCTCTATGCCAAC-3' and 5'-ATGGA-GCCACCGATCCACA-3').

### Aβ1−42 treatment

The synthetic Aβ1-42 peptide was purchased from Peptide Institute (Osaka, Japan), dissolved in 0.1% NH_3 _to the final concentration of 1 mM, and stored at -80°C until use. To confirm the state of the Aβ1-42 peptide, we performed Western blot analysis. Briefly, a stored Aβ1-42 peptide was subjected to 16% Tris-Tricine Gel (Invitrogen) electrophoresis and transferred to polyvinylidene difluoride (PVDF) membranes (Millipore, Billerica, MA). These membranes were incubated with a primary antibody against mouse monoclonal human Aβ (6E10; Covance, Emeyryville, CA). For detection, the membrane was incubated with a horseradish-peroxidase-conjugated Ig anti-mouse antibody. Immunoreaction signals were visualized with ECL™ or ECL Plus™ Western blotting detection reagent (GE Healthcare, Piscataway, NJ) and exposed to the LAS-3000 Mini Bio-imaging Analyzer System (FUJIFILM Co., Tokyo, Japan).

### Western blot analysis

The cells were washed with PBS and homogenized in lysis buffer (10 mM Tris-HCl (pH 7.4), 150 mM NaCl, 1 mM EDTA, 1% Triton X-100) containing a protease inhibitor cocktail (Roche, Mannheim, Germany). The homogenates were rocked at 4°C for 30 min and centrifuged at 13,000 × *g *at 4°C for 30 min to remove cell debris. The resulting supernatant was collected and protein concentration was determined using a BCA protein assay kit (Pierce, Rockford, IL). Equal amounts of protein were subjected to 7.5% or 5-20% gradient SDS polyacrylamide gel electrophoresis, and separated products were transferred to PVDF membranes. These membranes were then blocked with 5% skim milk in 10 mM Tris-HCl (pH 7.5), 150 mM NaCl, and 0.1% Tween 20 for 1 h at room temperature or overnight at 4°C. These membranes were incubated with primary antibodies, namely, the anti-ATBF1 (AT-6) antibody (1:1000; MBL, Nagoya, Japan), anti-p53 antibody (1:1000; Cell Signaling, Cambridge, UK), anti-ATM antibody (1:1000; Gene Tex, Irvine, CA), anti-ATM kinase pS1981 antibody (1:1000; Rockland, Gilbertsville, PA), or anti-actin antibody (1:2,000; Sigma, Saint Louis, MO). The membranes were washed, and then incubated with the appropriate secondary antibody conjugated to horseradish peroxidase. Immunoreaction signals were visualized with ECL™ or ECL Plus™ Western blotting detection reagent and exposed to the LAS-3000 Mini Bio-imaging Analyzer System. Signal intensity was determined using MultiGauge software (FUJIFILM).

### RNA interference

Endogenous ATBF1 was knocked down using predesigned Stealth™siRNA against ATBF1 (ATBF1 siRNA) and Stealth siRNA negative control (control siRNA) from Invitrogen (Carlsbad, CA). The ATBF1 siRNAs sequences are as follows: ATBF1-siRNA-1 sense (5'-UAC ACU GGU CAG ACC ACU GUC CUU G-3') and antisense (5'-CAA GGA CAG UGG UCU GAC CAG UGU -3'). ATBF1-siRNA-2 sense (5'- UAC ACU GGU CAG ACC ACU GUC CUU G-3') and antisense (5'-TAC ACT GGT CAG ACC ACT GTC CTT G-3'). The primary cultured neurons were transiently transfected with 50 nM ATBF1 siRNA or with control siRNA using Lipofectamine RNAiMAX (Invitrogen) in accordance with the manufacturer's instructions. The knockdown effects were examined after 48 h of incubation. The cultures were then processed for Western blot analysis, cell viability analysis and terminal deoxynucleotidyl transferase-mediated dUTP nick-end labeling (TUNEL) assay 16 h after Aβ1-42 treatment.

### Cell viability analysis

Neuronal viability was evaluated by CellTiter-Glo luminescent cell viability assay (Promega, Madison, WI), which is a method to determine the number of viable cells in culture based on the quantitation of ATP present, which indicates the presence of metabolically active cells. Briefly, primary cortical neurons were seeded onto poly-d-lysine-coated 96-well plates, and incubated for 72 h. For the ATBF1 knockdown experiment, the cells were transfected with ATBF1 siRNA or with control siRNA for 48 h as described above, cells were then treated with Aβ1−42, etoposide, or homocysteine at indicated doses for 16 h. After treatment, a volume of CellTiter-Glo Reagent was added to each well equal to the volume of cell culture medium. Then, the contents were mixed for 2 min on a shaker to induce cell lysis and the plates were incubated at room temperature for 10 min in the dark. Cellular luminescence intensity was measured using a GLOMAX 96-microplate luminometer (Promega).

### Plasmid constructs

The ATBF1 expression vector of an 11 kb full-length human cDNA [23] was inserted into the pCI vector (Promega) with an HA-tagged sequence at the 5'-terminus of the inserted sequence (HA-ATBF1) [25]. The 2.4 kb fragment upstream from the TATA-box of the human p21 (Waf1/Cip1) genomic fragment was subcloned into the basic luciferase reporter pGV-B vector (Toyo Ink Co., Ltd., Tokyo, Japan) [48].

### TUNEL assay

Apoptosis was assessed by TUNEL using an ApopTag Fluorescein Direct In Situ Apoptosis Detection kit in accordance with the manufacturer's instructions (Chemicon, Temecula, CA). Briefly, cells were fixed with 1% paraformaldehyde in PBS for 10 min at room temperature and permeabilized in EtOH:acetic acid (2:1) for 5 min at -20°C. Cells were then washed with PBS. Fluorescein-conjugated nucleotide and TdT enzyme were added to the cells, which were then incubated for 1 h at 37°C. Nuclei were stained with DAPI. Images were obtained using an AX70 fluorescence microscope (Olympus). The percentage of apoptotic cells was determined as the ratio of the number of DAPI-TUNEL-double-positive cells with respect to the total number of DAPI-positive cells. For the overexpression of ATBF1 in cultured cortical neurons, the neurons were transiently transfected with 0.5 μg HA-ATBF1 using FuGENE HD (Roche) in accordance with the manufacturer's instructions. Twenty-four hours after transfection, TUNEL was performed as described in above. After TUNEL, the neurons were incubated with the primary antibody against HA-tag (MBL) for 1 h at RT. The secondary antibody was Alexa-594-conjugated goat anti-rabbit IgG (Molecular Probes). Images were obtained using an AX70 fluorescence microscope (Olympus).

### Caspase-3/7 activity assay

Caspase-3/7 activity was assayed using a Caspase-Glo™ 3/7 assay kit (Promega), in accordence with the manufacturer's instructions. Briefly, primary cortical neurons were seeded on 96-well plates at a density of 1 × 10^6 ^cells/ml. After 3 days, the cells were treated with Aβ1-42 or DNA-damaging drugs. Caspase-Glo™ 3/7 reagent was then added to each well, and the plates were incubated at room temperature for 1 h. Cellular luminescence was measured using a GLOMAX 96-microplate luminometer (Promega).

### Immunoprecipitation

Primary cortical neurons were grown in 10 cm dishes. After reaching 50-70% confluence, the cells were treated with 10 μM Aβ1−42 or 1 μM etoposide for an indicated time. After incubation, the cells were washed twice with PBS, lysed in 1 ml of lysis buffer (10 mM Tris-HCl (pH 7.4), 150 mM NaCl, 1 mM EDTA, 1% Triton X-100, 50 mM NaF, and 100 uM sodium orthovanadate) containing protease inhibitor cocktail, and centrifuged at 13,000 × *g *at 4°C for 20 min. The resulting supernatant was immunoprecipitated overnight with a specific antibody against ATBF1 (AT-6) in the presence of protein G beads (Pierce) at 4°C. The immune complexes were washed four times with lysis buffer. The samples were subjected to 5-20% gradient SDS polyacrylamide ge electrophoresis, and separated products were transferred to a PVDF membrane and subjected to immunoblotting with a specific antibody against phosphorylated-ATM (pATM) at Ser 1981.

### X-ray irradiation and p21 promoter assay

ATM (+/+) and ATM (-/-) cells were transfected with p21 promoter-luciferase, pRL-TK-luciferase (as an internal control), and an indicated dose of the HA-ATBF1 vector or pCI-HA vector as the control using Lipofectamine 2000 (Invitrogen) in accordance with manufacturer's instructions. After 24 h, the cells were irradiated with X-ray at 2.5 Gy using a Softex M-80WE X-ray generator (SOFTEX, Japan) operating at 80 kv and 10 mA for 25 min with a copper shield. Nonirradiated dells were used as control. After 12 h, luciferase activity was measured using the Dual Luciferase Reporter Assay system (Promega) in accordance with the manufacturer's instructions.

### Statistical analysis

Statistical analysis was performed using a statistical package, GraphPad prism software (GraphPad Software, San Diego, CA). All values are presented as the mean ± SEM of at least three independent experiments.

## Abbreviations

ATBF1: AT-motif binding factor-1; Aβ: Amyloid-β peptide; AD: Alzheimer's disease; APP: Αmyloid precursor protein; PS: presenilin; APOE: apoplipoprotein E; ATM: Ataxia-telangiectasia mutated; pATM: phosphorylated ATM; PI3K: phospatidylinositol-3 kinase; TUNEL: Terminal deoxynucleotidyl transferase-mediated dUTP nick-end labeling.

## Competing interests

The authors declare that they have no competing interests.

## Authors' contributions

CGJ designed this study, carried out major parts of the experiments, and drafted the manuscript. KOU prepared primary cortical neurons. YM, TH, HH, and MJK carried out the experiments. KKK provided comments on the manuscript. MM participated in the design of the study and in drafting the manuscript.

All authors have read and approved the final manuscript

## Supplementary Material

Additional file 1 **Western blot analysis of Aβ1-42 peptide used in our experiments**. The stored Aβ1-42 peptide was diluted with culture medium to the final concentration of 5 μM, and then 0.5 (lane 1), 1 (lane 2), or 2.5 μl (lane 3) was loaded to 16% Tris-Tricine gel and probed with the monoclonal antibody 6E10 (recognizing residues 1-17 of Aβ). Click here for file

Additional file 2 **Effect of another ATBF1 siRNA (ATBF1 siRNA-2) on the viability of primary cortical neurons upon treatment with Aβ1-42**. A, Primary cortical neurons were transfected with ATBF1 siRNA-2 or control siRNA for 48 h. After transfection, the cells were then incubated in the presence or absence of 5 μM Aβ1-42 for 16 h. The expression levels of ATBF1 and actin were determined by Western blot analysis using the anti-ATBF1 and anti-actin antibodies. B, After transfection as described in Figure 3B, the cells were treated with or without 5 μM Aβ1-42 for 16 h. Cell viability was determined using a CellTiter-Glo luminescent cell viability assay kit and is shown as a percentage of surviving cells. All the values are presented as the mean ± SEM of three independent experiments. *p < 0.01 *vs *control siRNA treatment. N.S., not significant, as determined by Student's *t*-test. Click here for file

Additional file 3 **A, KU55933, a specific ATM inhibitor, shows a neuroprotective effect against Aβ 1-42-, etoposide-, and homocysteine-induced neurotoxicity**. Primary cortical neurons were seeded at a density of 1 × 10^6 ^cells/ml in poly-d-lysine-coated 96-well plates. Three days after plating, cells were pretreated with 0, 1, 5, or 10 μM KU55933 for 1 h, and subsequently treated for 16 h with 5 μM Aβ1-42, 1 μM etoposide (Etop), or 250 μM homocysteine (Hom). Cell viability was determined using a CellTiter-Glo luminescent cell viability assay kit. B, after transfection as described in Figure 3B, cells were pretreated for 1 h with or without 1 μM KU55933, and then cells were further incubated with or without 5 μM Aβ1-42 for 16 h. Cell viability was determined using a CellTiter-Glo luminescent cell viability assay kit. All the values are presented as the mean ± SEM of three independent experiments. *p < 0.001 *vs *control siRNA. N.S., not significant, as determined by one-way ANOVA followed by Duncan's test. Click here for file
