# Supplementary figures and images for: Beta-amyloid increases the expression level of ATBF1 responsible for death in cultured cortical neurons
Source: Mol Neurodegener. 2011 Jul 5;6:47. doi: 10.1186/1750-1326-6-47 (PMC3145572; doi:10.1186/1750-1326-6-47)

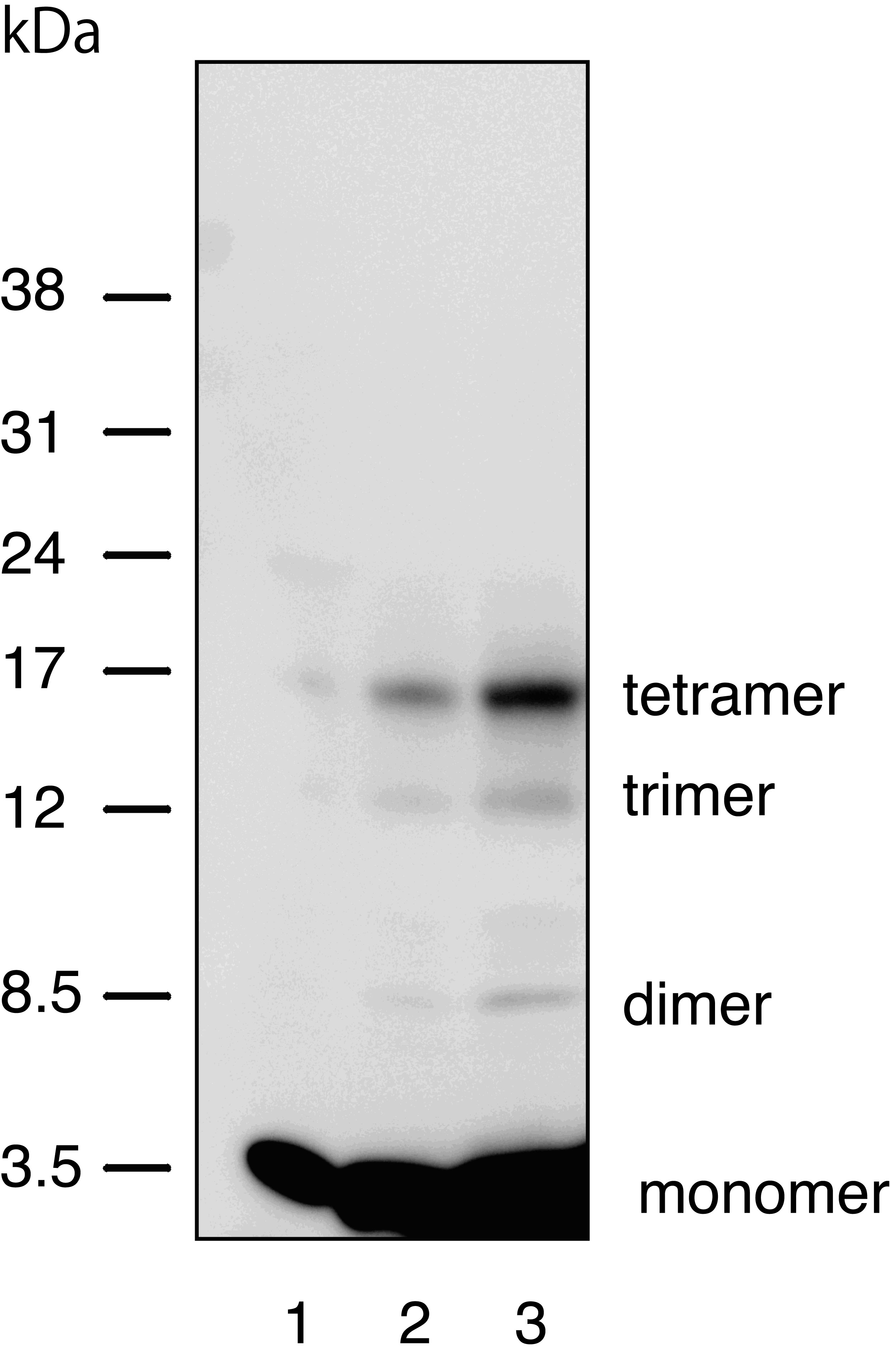

Supplement: Additional file 1 — Western blot analysis of Aβ1-42 peptide used in our experiments. The stored Aβ1-42 peptide was diluted with culture medium to the final concentration of 5 μM, and then 0.5 (lane 1), 1 (lane 2), or 2.5 μl (lane 3) was loaded to 16% Tris-Tricine gel and probed with the monoclonal antibody 6E10 (recognizing residues 1-17 of Aβ). [file 1750-1326-6-47-S1.JPEG]

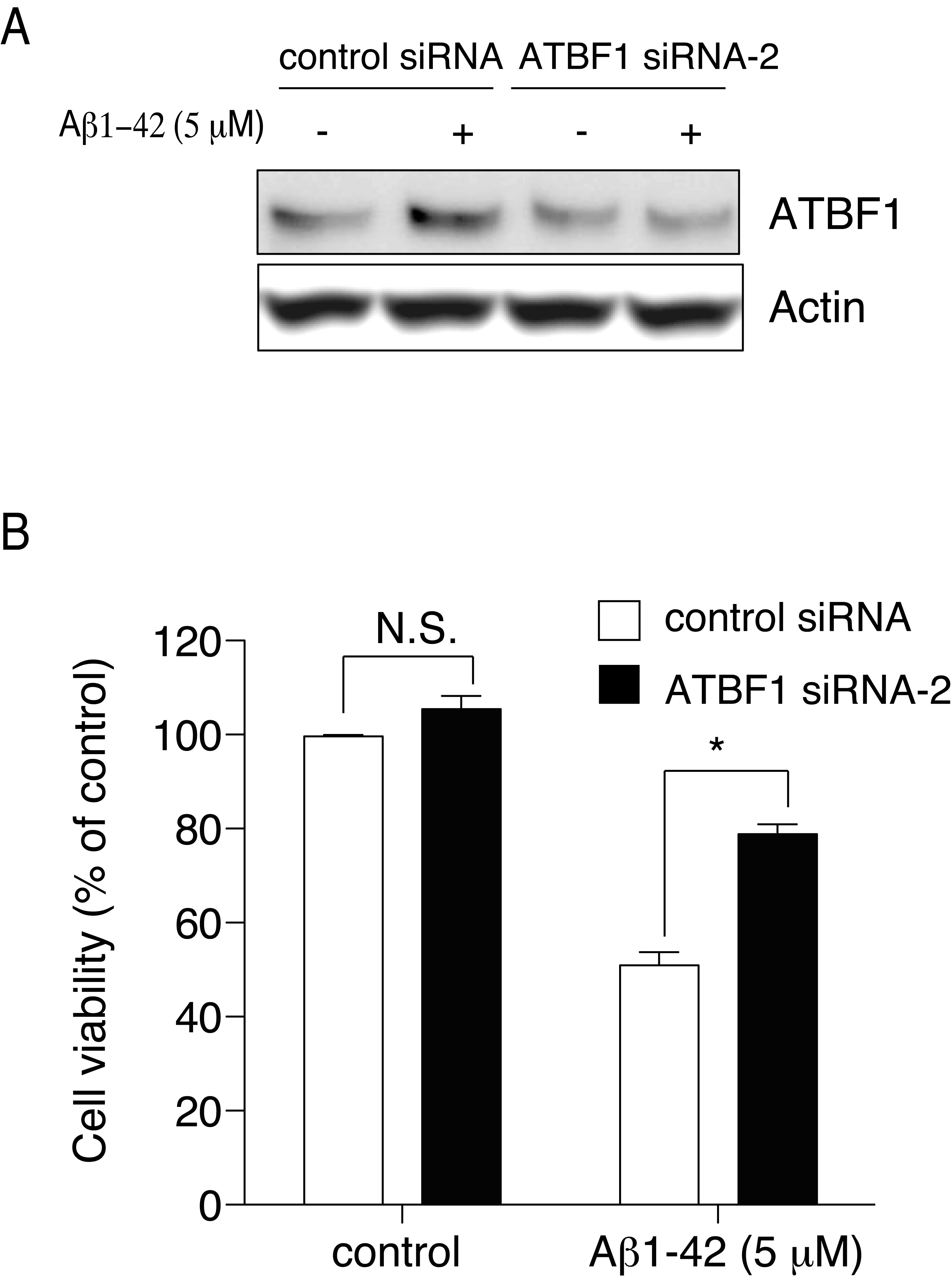

Supplement: Additional file 2 — Effect of another ATBF1 siRNA (ATBF1 siRNA-2) on the viability of primary cortical neurons upon treatment with Aβ1-42. A, Primary cortical neurons were transfected with ATBF1 siRNA-2 or control siRNA for 48 h. After transfection, the cells were then incubated in the presence or absence of 5 μM Aβ1-42 for 16 h. The expression levels of ATBF1 and actin were determined by Western blot analysis using the anti-ATBF1 and anti-actin antibodies. B, After transfection as described in Figure 3B, the cells were treated with or without 5 μM Aβ1-42 for 16 h. Cell viability was determined using a CellTiter-Glo luminescent cell viability assay kit and is shown as a percentage of surviving cells. All the values are presented as the mean ± SEM of three independent experiments. *p < 0.01 vs control siRNA treatment. N.S., not significant, as determined by Student's t-test. [file 1750-1326-6-47-S2.JPEG]

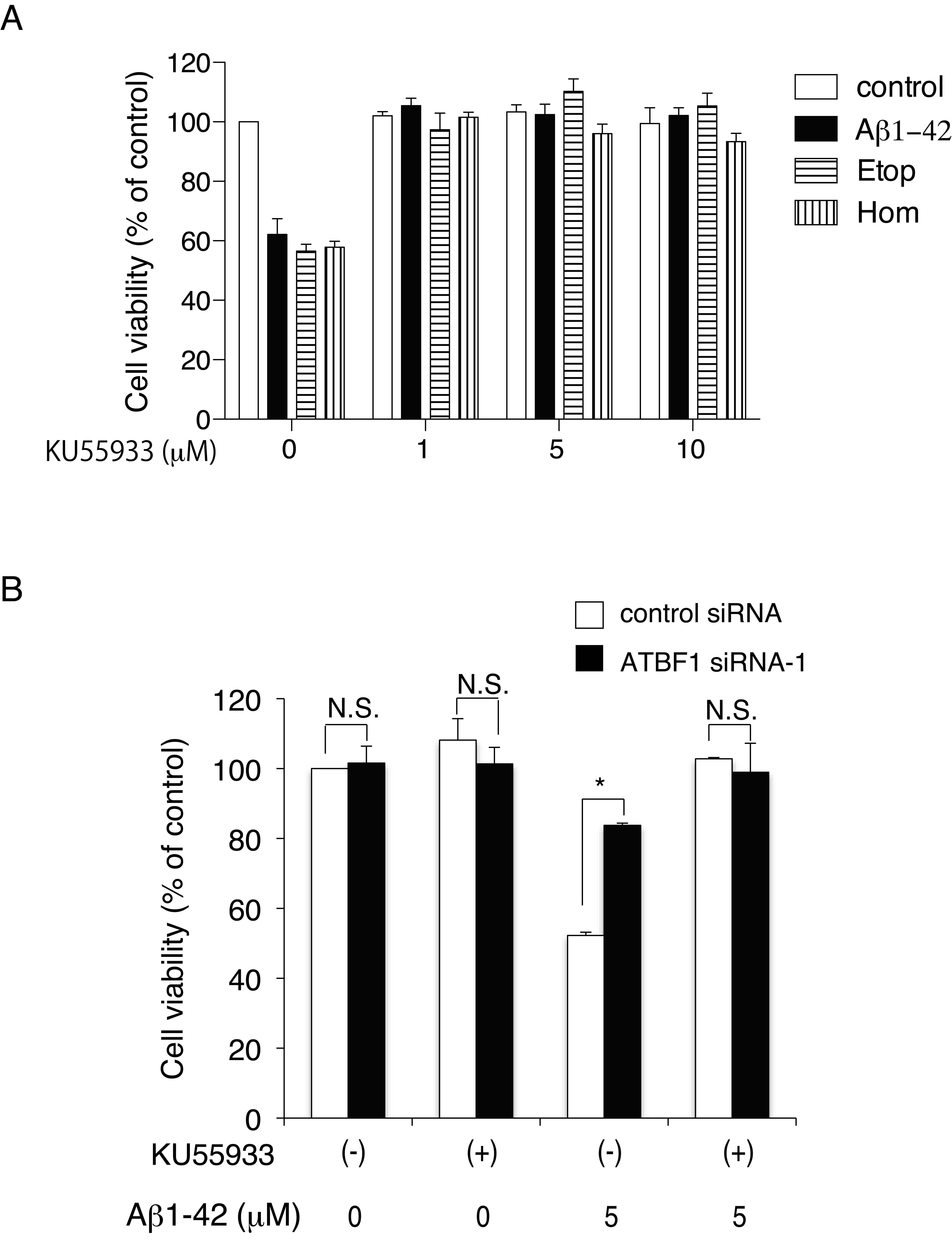

Supplement: Additional file 3 — A, KU55933, a specific ATM inhibitor, shows a neuroprotective effect against Aβ 1-42-, etoposide-, and homocysteine-induced neurotoxicity. Primary cortical neurons were seeded at a density of 1 × 10^6^ cells/ml in poly-d-lysine-coated 96-well plates. Three days after plating, cells were pretreated with 0, 1, 5, or 10 μM KU55933 for 1 h, and subsequently treated for 16 h with 5 μM Aβ1-42, 1 μM etoposide (Etop), or 250 μM homocysteine (Hom). Cell viability was determined using a CellTiter-Glo luminescent cell viability assay kit. B, after transfection as described in Figure 3B, cells were pretreated for 1 h with or without 1 μM KU55933, and then cells were further incubated with or without 5 μM Aβ1-42 for 16 h. Cell viability was determined using a CellTiter-Glo luminescent cell viability assay kit. All the values are presented as the mean ± SEM of three independent experiments. *p < 0.001 vs control siRNA. N.S., not significant, as determined by one-way ANOVA followed by Duncan's test. [file 1750-1326-6-47-S3.JPEG]
